# Knowledge and attitudes towards seizure first aid among attendees of Gateway Clinic in Bloemfontein

**DOI:** 10.4102/safp.v67i1.6208

**Published:** 2025-11-27

**Authors:** Ilias P. Masinde, Aileen M. Kamwendo, Desiree R. Thapo, Likhona Jaca, Lolwethu L. Qulu, Lungelo A. Gwala, Tebogo W. Kgasi, Joseph B. Sempa, Chika K. Egenasi

**Affiliations:** 1 Faculty of Health Sciences, University of the Free State, Bloemfontein, South Africa; 2 Department of Biostatistics, Faculty of Health Sciences, University of the Free State, Bloemfontein, South Africa; 3 Department of Family Medicine, Faculty of Health Sciences, University of the Free State, Bloemfontein, South Africa

**Keywords:** seizure, epilepsy, first aid, Gateway Clinic, Bloemfontein, knowledge, attitude

## Abstract

**Background:**

Providing first aid during an epileptic seizure can be lifesaving; however, many individuals lack the necessary knowledge and skills because of persistent misconceptions about epilepsy. This study assessed knowledge and attitudes towards seizure first aid among attendees of the Gateway Clinic in Bloemfontein.

**Methods:**

A cross-sectional analytical study was conducted using a structured questionnaire to evaluate participants’ knowledge and attitudes regarding seizure first aid.

**Results:**

Of the 466 questionnaires distributed, 391 were included in the study. Most participants were knowledgeable, with a median score of 13.0 (interquartile range [IQR]: 9.0–16.0). Attitudes towards epilepsy were positive, with a median score of 6.0 (IQR: 3.0–9.0). A majority, 64.7% of participants, believed that an object should be placed in the mouth during a seizure, and 33.5% were unsure whether epilepsy was contagious.

**Conclusion:**

Participants at the Gateway Clinic were knowledgeable and had positive attitudes towards epilepsy and seizure first aid. Nonetheless, misconceptions persist, particularly regarding harmful practices during seizures. Public education and structured training on seizure first aid remain essential to improve safety and awareness.

**Contribution:**

This study highlights the need to strengthen community awareness and education on epilepsy and seizure first aid to reduce stigma and enhance appropriate responses during seizure events.

## Introduction

Epilepsy is a chronic neurological condition that affects numerous people of all ages in Africa and worldwide. Epilepsy accounts for a significant portion of the world’s disease burden, affecting around 50 million people worldwide.^1^ In 2019, the global prevalence of epilepsy was 682.7 per 100 000, with 52.5 million recorded cases.^2^ This is an 11.2% increase from 2010 to 2019.^3^ More than five million people are diagnosed with epilepsy every year; this figure is expected to increase.^3^ The prevalence rate is higher in males than in females, although the difference is not substantial.^2^

The causes of epilepsy are diverse and include structural abnormalities, genetic factors, infections, stroke, tumours and metabolic disorders. Despite advances in medical research, the underlying cause remains unknown in about 50% of cases globally. Importantly, epilepsy is not a contagious condition.^1^

The overall lifetime prevalence of epilepsy is 7.6 per 1000 population, being higher in low- and middle-income countries (LMICs) (8.75 per 1000).^3^ The worldwide point prevalence of active epilepsy is 6.38 per 1000 persons and between 5.57 and 7.30 per 1000 in LMICs.^3^ This exemplifies the higher burden in LMICs.^3^ Around 7.6 per 1000 persons will have epilepsy in their lifetime.^3^ Low- and middle-income countries have a higher incidence of epilepsy compared to high-income countries (HICs), with the incidence rates being 139 per 100 000 and 48.9 per 100 000, respectively.^3^ This could be attributed to the increased risk of endemic conditions such as malaria, the higher incidence of road traffic injuries and the limited availability of preventative health programmes and accessible care, among other factors.^1^ According to the World Health Organization (WHO), approximately 80% of people with epilepsy live in LMICs.^1^

While epilepsy is a complex disease, it is manageable and controllable,^1^ and 70% of people living with epilepsy could be seizure-free with the appropriate use of antiseizure medicines.^1^ Even with the favourable prognosis of epilepsy, there exists a significant barrier to treatment as the availability of generic antiseizure medicines in the public sector of LMICs is less than 50%.^1^

In low-income countries, early death among people with epilepsy is significantly higher, and this can be attributed to lack of access to health facilities and preventable causes such as burns, head injuries and drowning.^3^ This can be mitigated by educating the public about the risk of death associated with epilepsy and improved access to treatments.^3^

About 75% of people with epilepsy have had their first seizure before the age of 20 years.^4^ The prevalence of epilepsy in sub-Saharan Africa is estimated to be nine per 1000 persons for active epilepsy, and for lifetime epilepsy, the prevalence is estimated to be 16 per 1000 persons.^5^ This means that there are fewer people with active epilepsy than the total number of those affected.^5^ South Africa is estimated to have an incidence rate of 25 per 100 000 per year.^6^ Epilepsy South Africa estimates that epilepsy affects about one in 100 South Africans.^7^

A seizure is a sudden, uncontrolled burst of electrical activity in the brain.^8^ First aid is the first and immediate help given to an individual who is ill or has a physical injury before obtaining professional medical assistance.^9^ Unifying the two, seizure first aid is defined as a helpful action taken by people witnessing a patient experience an epileptic seizure. The first aid management of epileptic seizures is based on the knowledge of what to do when a person is experiencing a seizure. This involves keeping the individual having the seizure as safe as possible to prevent secondary injuries that may arise from the seizure.^10^ Although these seizures may be scary, they are not always emergencies.^10^ First aid management often works in these scenarios, but in instances where it does not, it is essential to keep performing the actions while waiting for the ambulance to arrive.^11^

To prevent injury during a seizure, potentially harmful objects and furniture should be moved away from the person having a seizure.^7^ Something soft should be placed under the head of the person having a seizure, especially if they are on the floor.^7^ Their head should be turned to the side to maintain a patent airway. Actions such as restraining the person or placing anything in their mouth during the seizure are prohibited.^7^ Individuals experiencing a seizure should not be left unsupervised until their full recovery is ensured.^7^

The execution of first aid management techniques determines the state of the person after they recover from the seizure.^12^ It is, therefore, imperative to ensure that the appropriate criteria are meticulously followed to ensure that the patient recovers adequately from the seizure, as the individual could easily suffer from a low oxygen level in the blood, and this can be life-threatening.^13^ Providing seizure first aid information and training for the public may also assist in keeping members of the public informed about what to do when someone around them has a seizure; this could save lives.

A study by Kateb et al. in Al-Madinah City, Saudi Arabia, found that most citizens had limited knowledge of seizure first aid and could not respond during emergencies. Higher levels of education and employment were associated with better understanding, highlighting the importance of targeted epilepsy education programmes to improve public awareness and readiness.^14^

Kolahi et al. assessed primary school teachers’ knowledge, attitudes and first aid practices in Iran. While many teachers had witnessed seizures in public and were familiar with some first-aid measures, most also expressed positive attitudes towards individuals with epilepsy. The authors also recommended implementing epilepsy training programmes for teachers.^15^

There are misconceptions and myths regarding the causes of epilepsy and epileptic seizures among certain members of the public based on their religious or spiritual beliefs.^16^ This can affect the attitude of members of the public and prevent them from offering assistance or providing first aid to a person having a seizure.

Javed et al. investigated common myths and misconceptions about epilepsy in Pakistan. They found that most participants relied on informal sources, mainly friends and family, for information, with few receiving formal education on the topic. Spiritual and religious beliefs strongly shaped their understanding. The study highlighted the need for targeted educational initiatives, recommending social media as a tool to raise awareness, correct misconceptions and reduce stigma.^17^

Bones and Dein highlighted in their study that the association between spirituality and epilepsy remains strong across many cultures. In some communities, epilepsy is interpreted as a manifestation of witchcraft, demonic possession, poisoning or even a contagious condition, often leading to the belief that it requires spiritual or traditional healing rather than medical intervention.^16^

In Ethiopia, epilepsy is often perceived through cultural metaphors linking it to evil spirits, divine punishment and witchcraft, which influence health-seeking behaviours and lead to traditional practices such as inhaling smoke from matchsticks or using holy water for treatment.^18^ Similarly, Museka et al. found that in rural Limpopo and Mpumalanga, caregivers and family members often held negative attitudes towards people with epilepsy, with many believing they should not marry. These beliefs contribute to stigma and can negatively impact the well-being of individuals living with epilepsy.^19^

Eze et al. demonstrated the effectiveness of an epilepsy-specific intervention in Nigeria by evaluating the impact of structured health education on trainee teachers. The study showed significant improvements in knowledge, attitudes and first aid practices following the intervention.^20^ Including videos and pamphlets further enhanced learning, highlighting their value in epilepsy education and seizure first aid training.^21^

Although the studies above have exemplified the misconceptions held about epilepsy and the limited knowledge thereof, there has not been a study to investigate the knowledge about seizure first aid and attitudes of the public towards epilepsy in Bloemfontein, South Africa.

Hence, this study aimed to assess the knowledge gap among public members by investigating their knowledge and attitudes towards seizure first aid among attendees of the Gateway Clinic in Bloemfontein. Furthermore, no published study has investigated the knowledge and attitudes towards seizure first aid in Bloemfontein, South Africa.

## Research methods and design

This study was a cross-sectional, analytical, questionnaire-based study.

### Study setting

The study was conducted in the Mangaung metropolitan municipality of Bloemfontein, central South Africa. There are 41 Primary health care clinics in Mangaung.^22^ One of these clinics, the Gateway Clinic, was used for this study. This clinic was chosen because it is located in the centre of Bloemfontein and caters for a large, diverse population of patients.

### Study population

This study population consisted of adults attending the Gateway Clinic in Bloemfontein during the study period who were willing to provide informed consent to participate. Gateway Clinic sees approximately 1987 patients per month and 19 656 patients annually. Relatives and caregivers often accompany these patients.

### Inclusion criteria

Participants attending the Gateway Clinic who were 18 years old and above consented to participate in the study.

### Exclusion criteria

All medical personnel of Gateway Clinic, persons who were too ill and required urgent medical attention and patients who did not consent to participate in the study were excluded from the study.

### Sampling method and size

A convenience sampling method was used for this study. The calculated sample size for the study was 377, based on a 95% confidence interval and a 5% margin of error using the Raosoft sample size calculator. However, 466 participants were recruited to enhance the study’s robustness, and 391 were included. The study period was from August to October 2024. A total of 391 of the 466 questionnaires collected were used in the study; 75 were not used because of incompleteness. The response rate for the study was 83.9%.

### Data collection

Data were collected at the Gateway Clinic during the operational hours (08:00 to 16:00) on weekdays between August and October 2024. Researchers identified eligible participants based on the study’s inclusion criteria. Participants were approached in the waiting area, provided with detailed information about the study, and informed consent was obtained prior to participation. The participant’s right to withdraw or refuse consent was duly explained to each participant. Questionnaires were completed while participants waited for consultation with health care providers. To accommodate the region’s linguistic diversity, questionnaires were translated by knowledgeable translators and made available in English, Afrikaans and Sesotho, the predominant local languages. Illiterate participants were discreetly removed from the queue to ensure privacy, and questions were read out to them without further explanations to avoid biased answers. All responses were anonymous.

The data were recorded on REDCap, a data management software, within the same week of collection. The researchers reviewed the questionnaires for completeness. Incomplete questionnaires were not captured. Each questionnaire entry had a unique identification number to prevent duplication.

The questionnaire was developed using relevant information from the literature on epilepsy seizure first aid. Its content validity and contextual relevance were reviewed and confirmed by family physicians knowledgeable in epilepsy to ensure its suitability for the study.

The questionnaires were structured as follows:

- Section A: Demographic information.
- Section B: Additional information.
- Section C: Knowledge about epilepsy.
- Section D: Knowledge about seizure first aid.
- Section E: Attitudes towards epilepsy.

Participants’ knowledge was measured using a system adopted from a knowledge, attitudes and practices (KAP) study on Type 2 Diabetes by Khan et al.^23^

The demographic information in Section A included age, gender, education, religion, marital status and employment of the participants. Sections B, C and D had questions requiring either ‘True’, ‘False’ or ‘I don’t know’ answers. Answering with ‘I don’t know’ was considered incorrect. Correct answers were awarded 1 mark, and incorrect answers were not awarded any marks. The completed questionnaires were marked, and the participants were graded as follows, based on their total score:

- Very knowledgeable (20-26).
- Knowledgeable (13-19).
- Average to below average knowledge (6-12).
- Little to no knowledge (0-5).

Section E used a 5-point Likert scale to assess participants’ attitudes towards seizure first aid. The −2 to 2 Likert scale gave each question a mark.

For selected questions (1–9) (positive statements), ‘strongly agree’ was awarded 2 marks, ‘Agree’ was awarded 1 mark, ‘Neutral’ was awarded 0 marks, ‘disagree’ was awarded –1 mark and ‘strongly disagree’ was awarded –2 marks. For questions 10 and 11 (the reverse scored questions), ‘strongly agree’ was awarded –2 marks, ‘Agree’ was awarded −1 mark, ‘Neutral’ was awarded 0 marks, ‘disagree’ was awarded 1 mark and ‘strongly disagree’ was awarded 2 marks. The total mark was calculated by adding the marks from all the questions. After analysis, the results were used to grade the participants’ attitudes in the following categories:

Positive attitude (score between 1 and 22), neutral attitude (score of 0) and negative attitude (score between –1 and –22).

### Pilot study

Following ethical approval from the Health Sciences Research Ethics Committee (HSREC) of the University of the Free State, a 2-day pilot study was conducted at the Gateway Clinic to test the data collection tool. Eleven questionnaires were completed. The study biostatistician was given restricted collaborator access on REDCap to download and analyse the pilot data. Findings from the pilot led to revisions of the tool, and the pilot data were excluded from the final study sample.

### Data analysis

Responses on the data collection sheet were numerically coded to facilitate seamless transcription into Microsoft Excel, ensuring accurate and efficient data entry. The completed questionnaire data were captured and securely stored on the REDCap platform. The University of the Free State biostatistician performed the required statistical analyses.

All statistical analyses were conducted using R software, version 4.4.2 (R Foundation for Statistical Computing, Vienna, Austria). Descriptive statistics were used to summarise the data, including cumulative frequencies and percentages for categorical variables, and medians for continuous variables. For clarity, results are presented using both tables and graphical formats.

To assess associations between variables, appropriate non-parametric and categorical tests were applied. The Kruskal–Wallis rank sum test was used to compare continuous variables with levels of knowledge and attitudes. Fisher’s exact test was used for categorical variables to evaluate associations with knowledge and attitude levels. A *p*-value of < 0.05 was considered statistically significant.

### Ethical considerations

Approval was obtained from the HSREC of the University of the Free State (UFS-HSD2024/0381/2307-0002) before commencing the research. All participants’ identification was protected by assigning each participant a code for all documents and questionnaires to ensure confidentiality. The researcher kept all paper-based information in a secure location, accessible only to those involved in the study.

## Results

Of the 466 questionnaires distributed to participants, 391 were included in the study. Seventy-five questionnaires were excluded because of being incomplete. The majority of participants were female (77.0%), with a median age of 24 years (see Table 1 for additional demographic information).

**TABLE 1:** Demographic information of participants.

Characteristics	*n*	%
**Age (years)**		
18–19	33	8.4
20–29	228	58.3
30–39	68	17.4
40–49	32	8.2
50–59	17	4.3
60–69	10	2.6
70–79	2	0.5
80–89	1	0.3
**Gender**		
Female	301	77.0
Male	90	23.0
Other	0	0.0
**Education**		
Grade 1–6	5	1.3
Grade 7–12	156	33.9
Tertiary	227	58.1
No formal education	3	0.8
**Marital status**		
Single	302	77.2
Married	55	14.1
Divorced	13	3.3
Widowed	9	2.3
Prefer not to say	12	3.1
**Employment status**		
Employed	74	18.9
Unemployed	93	23.8
Student	205	52.4
Pensioner	11	2.8
Other	8	2.0
**Religious status**		
Christianity	376	96.2
Islam	4	1.0
Judaism	0	0.0
Buddhism	0	0.0
Hinduism	0	0.0
Other	11	2.8

Note: Table 1 shows that the majority of participants in this study were single, female, had attained tertiary education, and identified as Christians.

Most participants were knowledgeable, with a median score of 13.0 (interquartile range [IQR]: 9.0–16.0) from a range of 0.0 to 23.0 (see Table 3 for detailed knowledge related questions and corresponding responses).

**TABLE 2:** The number of participants who received seizure first aid training and where they received their training.

Characteristics	*n*	%
**Do you have epilepsy?**		
Yes	10	2.6
No	351	89.8
I do not know	30	7.7
**Do you know someone with epilepsy?**		
Yes	111	28.4
No	255	65.2
I do not know	25	6.4
**Have you had seizure first aid training?**		
Yes	43	11.0
No	339	86.7
I do not know	9	2.3
**Where did you learn about seizure first aid training?**		
Books	3	0.8
Television, radio, newspaper or journal	3	0.8
Internet	8	2.0
Relatives	3	0.8
Friends	1	0.3
Teachers	8	2.0
Medical staff	16	4.1
Do not remember	2	0.5
Other	3	0.8

Note: Table 2 indicates that only a small number of participants had received seizure first aid training; most of them were trained by medical staff.

**TABLE 3:** Participants’ knowledge of epilepsy.

No.	Knowledge questions	Yes		No		I do not know	
		*n*	%	*n*	%	*n*	%
1	Epilepsy is a neurological disorder.	**203**	**51.9**	31	7.9	157	40.2
2	Falling, urinating yourself and shouting are symptoms of epileptic seizures.	**181**	**46.3**	74	18.9	136	34.8
3	There are different types of seizures.	**214**	**54.7**	17	4.3	160	40.9
4	Epilepsy is curable.	72	18.4	**137**	**35.0**	182	46.5
5	Most seizures are controlled by regular drug therapy.	**217**	**55.5**	25	6.4	149	38.1
6	Epilepsy is hereditary.	**104**	**26.6**	55	14.1	232	59.3
7	Foaming of the mouth, whole body shaking and loss of consciousness are symptoms of epileptic seizures.	**301**	**77.0**	11	2.8	79	20.2
8	Insanity, witchcraft and spiritual attacks are causes of epilepsy.	33	8.4	**234**	**59.8**	124	31.7
9	Curses, bad blood and poisoning are causes of epilepsy.	27	6.9	**188**	**48.1**	176	45.0
10	Epilepsy is contagious.	54	13.8	**206**	**52.7**	131	33.5
11	Should you restrain a person that is experiencing a seizure?	125	32.0	**128**	**32.7**	138	35.3
12	Seizure first aid includes moving objects away from someone experiencing a seizure.	**259**	**66.2**	27	6.9	105	26.9
13	Measuring the duration of the seizure is important.	**269**	**68.8**	12	3.1	110	28.1
14	I should try to open the mouth of a seizing individual and put something in between their jaw.	253	64.7	**35**	**9.0**	103	26.3
15	I should rub oil on a person experiencing a seizure.	27	6.9	**117**	**29.9**	247	63.2
16	I should sprinkle water on the face of a seizing person to try to wake them up.	157	40.2	**81**	**20.7**	153	39.1
17	Staying with the seizing individual until the ambulance arrives is important.	**318**	**81.3**	13	3.3	60	15.3
18	I should put the legs of a person experiencing a seizure in fire.	15	3.8	**271**	**69.3**	105	26.9
19	I should not prevent the seizing individual from falling.	**116**	**29.7**	156	39.9	119	30.4
20	When someone starts experiencing a seizure, I should start praying.	**132**	**33.8**	153	39.1	106	27.1
21	I should carefully roll the seizing individual to their side.	**212**	**54.2**	32	8.2	147	37.6
22	I should do nothing to a person experiencing a seizure and just wait for it to stop.	57	14.6	**215**	**55.0**	119	30.4
23	I should run away from a person experiencing a seizure.	5	1.3	**357**	**91.3**	29	7.4
24	Do you think it is appropriate to take a seizing patient to a sangoma or pastor?	64	16.4	**222**	**56.8**	105	26.9
25	Should you take a seizing patient to the hospital?	**349**	**89.3**	10	2.6	32	8.2

Note: Table 3 outlines participants’ responses to the knowledge question. The majority erroneously believe that inserting objects into the mouths of individuals experiencing seizures is appropriate, while one-third either resort to prayer or are unsure about the correct approach. The values in bold indicates the correct answers.

No., number.

Most attendees of the Gateway Clinic had a positive attitude towards epilepsy, with a median score of 6.0 (IQR: 3.0, 9.0), from a range of −14.0 to 16.0 (see Table 4 for detailed attitude-related questions and responses).

**TABLE 4:** Attitudes of participants to epilepsy.

No	Attitude questions	Strongly agree		Agree		Neutral		Disagree		Strongly disagree	
		*n*	%	*n*	%	*n*	%	*n*	%	*n*	%
1	People with epilepsy should notify their partners before marriage.	251	64.2	106	27.1	19	4.9	8	2.0	7	1.8
2	Children with epilepsy can attend normal schools.	159	40.7	147	37.6	53	13.6	22	5.6	10	2.6
3	People with epilepsy can have a normal life.	187	47.8	151	38.6	35	9.0	14	3.6	4	1.0
4	People with epilepsy can have children.	204	52.2	134	34.3	37	9.5	12	3.1	4	1.0
5	People with epilepsy can achieve high levels of education.	241	61.6	116	29.7	22	5.6	9	2.3	3	0.8
6	I am afraid of being alone with someone with epilepsy.	55	14.1	82	21.0	104	26.6	89	22.8	61	15.6
7	As an employer, I would hire someone with epilepsy.	114	29.2	153	39.1	88	22.5	25	6.4	11	2.8
8	People with epilepsy can do sport.	91	23.3	129	33.0	118	30.2	40	10.2	13	3.3
9	I would not be embarrassed if someone in my family had epilepsy.	250	63.9	83	21.2	21	5.4	14	3.6	23	5.9
10	Children with epilepsy should be sent away to a special school.	22	5.6	53	13.6	94	24.0	113	28.9	109	27.9
11	People with epilepsy should not marry.	19	4.9	16	4.1	18	4.6	111	28.4	227	58.1

Note: Table 4 shows that the majority of the participants agree that people with epilepsy can attend normal schools and live a normal life.

## Discussion

The study explored the knowledge and attitudes of attendees of the Gateway Clinic towards seizure first aid in Bloemfontein. Most of the study participants were female, consistent with findings from other studies on seizure first aid.^24^ The majority of our participants were between 20–29-years-old, which is consistent with the findings reported in similar studies.^24^ The young age group may be because of the clinic being in the centre of town and near various tertiary institutions, which may also be the reason for a high number of participants who are single and have tertiary education. Participants were predominantly female, a finding that aligns with those from other studies.^24^ This may be attributed to the study being conducted at a primary health care facility that offers paediatric and antenatal care services. Most of our participants neither had epilepsy nor knew someone with the condition and had not received any seizure first aid training (also see Table 2). Findings are consistent with those reported by Al-Hayani et al.,^25^ where most of their study population had neither received first aid training nor witnessed an epileptic seizure. Also, their study participants denied a personal history of epilepsy.^25^ This may be because of a lack of awareness of the neurological disorder in the community. The few participants who had learned about seizure first aid mainly had received this information from medical staff. This finding is consistent with those of Eze et al., who reported that medical personnel play a crucial role in enhancing first aid management skills related to epilepsy.^20^

### Knowledge

More than half of the participants correctly identified epilepsy as a neurological disorder, which aligns with findings reported by Kateb et al., where the majority of their study population recognised epilepsy as a neurological disorder.^14^ More than half of the participants were also aware of different types of seizures. This is important for recognising and responding appropriately to different types of seizures, including those that are not easily noticeable, such as absence seizures,^26^ particularly in public settings where emergency health care may not be readily accessible. Similarly, a previous study also found that most participants were aware of the various types of their seizures.^27^

Most participants identified foaming at the mouth, shaking and loss of consciousness as symptoms of epilepsy, likely because these are the most observed seizure symptoms and are frequently portrayed in the media.^28^ This is consistent with findings reported by Eze et al.^20^ Less than half of the participants recognised falling, involuntary urination and shouting as symptoms of epilepsy. Similarly, fewer than half believed that epilepsy is curable, while more than half correctly acknowledged that it is not contagious. These findings are consistent with those reported by Kateb et al.^14^ A small percentage of their study population thought that epilepsy was contagious. The majority agree that epilepsy can be controlled with medication.

Most also recognised the importance of moving objects away from a person having a seizure, rolling them onto their side, timing the seizure and staying with them until help arrives. These findings are supported by Hakami et al.^24^ The majority of their study population would roll the individual experiencing a seizure to their side and clear the area surrounding them.

Consistent with findings by Gugssa and Haidar, a significant minority of participants still hold cultural and religious misconceptions, including the belief that seizures are caused by witchcraft.^18^ This highlights how deeply cultural and religious beliefs influence people’s attitudes, values and daily lives. Some participants were uncertain about the harmful practice of placing the legs of a person having a seizure into fire. Similar uncertainty was reported by Eze et al.^20^ This practice is incorrect and dangerous. This highlights the importance of engaging religious and cultural leaders who possess the appropriate knowledge and attitudes regarding epilepsy and seizure first aid, given their influential role in their communities. The need for this is underscored by the fact that many participants were unsure or indicated they would take a person experiencing a seizure to a pastor, sangoma, or rely solely on prayer. These findings are consistent with those of a similar study conducted in Ethiopia.^18^

The misconception of placing an object in the mouth of someone having a seizure was widespread among participants. This belief, often rooted in the desire to prevent tongue-biting, reflects a lack of accurate knowledge about seizure first aid and has been reported in studies from Ethiopia^18^ and Saudi Arabia.^24^ However, this practice can cause more harm than good, underscoring the need for targeted public education to correct such harmful misunderstandings. Encouragingly, most participants supported taking a person experiencing a seizure to the hospital, a finding also reported by Eze et al.^20^

A statistically significant relationship was observed between participants’ knowledge scores and age, with a general trend of increasing knowledge scores as age increased (also see Table 5). This aligns with findings from other studies, which suggest that older individuals may have had greater exposure to experiences that enhance their understanding of epilepsy and seizure first aid. For instance, a study on first aid knowledge among undergraduate health students in Riyadh, Saudi Arabia, found that older students, particularly those in advanced stages of their studies, exhibited higher knowledge scores than their younger counterparts.^29^ Similarly, research among hospital staff in Henan, China, demonstrated that age, educational status and professional experience significantly correlated with seizure first aid knowledge, with older staff members displaying higher levels of understanding.^30^

**TABLE 5:** Association between participants’ demographics, knowledge and attitude scores category.

No	Demographic items	Knowledge categories								*p*	Attitude categories						*p*
		Little to no knowledge		Average to below average knowledge		Knowledgeable		Very knowledgeable			Negative attitude		Neutral attitude		Positive attitudes		
		*n*	%	*n*	%	*n*	%	*n*	%	*n*	%	*n*	%	*n*	%
1	**Age (years)**	26	21.37	22	20.3	24	21.32	30	25.41	0.008	26	20.32	28	22.40	24	21.33	0.600
2	**Gender**	-	-	-	-	-	-	-	-	0.800	-	-	-	-	-	-	0.150
	Male	10	23.3	29	22.5	46	24.6	5	15.6	-	5	18.5	0	0.0	85	24.1	-
	Females	33	76.7	100	77.5	141	75.4	27	84.4	-	22	81.5	11	100.0	268	75.9	-
3	**Highest level of education**	-	-	-	-	-	-	-	-	0.073	-	-	-	-	-	-	0.120
	Grade 1–6	0	0.0	1	0.8	4	2.1	0	0.0	-	0	0.0	0	0.0	5	1.4	-
	Grade 7–12	14	32.6	60	46.5	65	34.8	17	53.1	-	11	40.7	4	36.4	141	39.9	-
	Tertiary	27	62.8	68	52.7	117	62.6	15	46.9	-	14	51.9	7	63.6	206	58.4	-
	No formal education	2	4.7	0	0.0	1	0.5	0	0.0	-	2	7.4	0	0.0	1	0.3	-
4	**Marital status**	-	-	-	-	-	-	-	-	0.200	-	-	-	-	-	-	0.300
	Single	29	67.4	106	82.2	145	77.5	22	68.8	-	20	74.1	7	63.6	275	77.9	-
	Married	11	25.6	16	12.4	21	11.2	7	21.9	-	5	18.5	2	18.2	48	13.6	-
	Divorced	0	0.0	3	2.3	8	4.3	2	6.3	-	0	0.0	1	9.1	12	3.4	-
	Widowed	1	2.3	2	1.6	5	2.7	1	3.1	-	0	0.0	0	0.0	9	2.5	
	Prefer not to say	2	4.7	2	1.6	8	4.3	0	0.0	-	2	7.4	1	9.1	9	2.5	-
5	**Employment status**	-	-	-	-	-	-	-	-	0.600	-	-	-	-	-	-	0.300
	Employed	11	25.6	20	15.5	32	17.1	11	34.4	-	6	22.2	4	36.4	64	18.1	-
	Unemployed	8	18.6	33	25.6	43	23.0	9	28.1	-	8	29.6	5	45.5	80	22.7	-
	Student	22	51.2	74	57.4	99	52.9	10	31.3	-	12	44.4	2	18.2	191	54.1	-
	Pensioner	1	2.3	1	0.8	8	4.3	1	3.1	-	1	3.7	0	0.0	10	2.8	-
	Other	1	2.3	1	0.8	5	2.7	1	-	-	0	0.0	0	0.0	8	2.3	-
6	**Religion**	-	-	-	-	-	-	-	-	-	-	-	-	-	-	-	0.600
	Christianity	41	95.3	123	95.3	181	96.8	31	96.9	0.600	26	96.3	11	100.0	339	96.0	-
	Islam	0	0.0	2	1.6	1	0.5	1	3.1	-	0	0.0	0	0.0	4	1.1	-
	Judaism	0	0.0	0	0.0	0	0.0	0	0.0	-	0	0.0	0	0.0	0	0.0	-
	Buddhism	0	0.0	0	0.0	0	0.0	0	0.0	-	0	0.0	0	0.0	0	0.0	-
	Hinduism	0	0.0	0	0.0	0	0.0	0	0.0	-	0	0.0	0	0.0	0	0.0	-
	Other	2	4.7	4	3.1	5	2.7	0	0.0	-	1	3.7	0	0.0	10	2.8	-
7	**Do you have epilepsy?**	-	-	-	-	-	-	-	-	**< 0.001**	-	-	-	-	-	-	**0.024**
	Yes	0	0.0	5	3.9	4	2.1	1	31.0	-	1	3.7	0	0.0	9	2.5	-
	No	33	76.7	111	86.0	176	94.1	31	96.9	-	20	74.1	9	81.8	322	91.2	-
	I do not know	10	23.3	13	10.1	7	3.7	0	0.0	-	6	22.2	2	18.2	22	6.2	-
8	**Do you know someone with epilepsy?**	-	-	-	-	-	-	-	-	**< 0.001**	-	-	-	-	-	-	0.100
	Yes	8	18.6	24	18.6	66	35.3	13	40.6	-	6	22.2	4	36.4	101	28.6	-
	No	28	65.1	94	72.9	114	61.0	19	59.4	-	16	59.3	6	54.5	233	66.0	-
	I do not know	7	16.3	11	8.5	7	3.7	0	0.0	-	5	18.5	1	9.1	19	5.4	-
9	**Have you had seizure first aid training?**	-	-	-	-	-	-	-	-	**< 0.001**	-	-	-	-	-	-	0.200
	Yes	2	4.7	11	8.5	20	10.7	10	31.3	-	1	3.7	2	18.2	40	11.3	-
	No	38	88.4	116	89.9	165	88.2	20	62.5	-	24	88.9	9	81.8	306	86.7	-
	I do not know	3	7.0	2	1.6	2	1.1	2	6.3	-	2	7.4	0	0.0	7	2.0	-
10	**Where did you learn about seizure first aid?**	-	-	-	-	-	-	-	-	-	-	-	-	-	-	-	-
	Books	1	2.3	0	0.0	1	0.5	1	3.1	0.100	0	0.0	0	0.0	3	0.8	> 0.900
	Television, radio, newspaper or journal	0	0.0	1	0.8	1	0.5	1	3.1	0.400	0	0.0	0	0.0	3	0.8	> 0.900
	Internet	0	0.0	3	2.3	4	2.1	1	3.1	0.800	0	0.0	1	9.1	7	2.0	0.300
	Doctors or nurses	0	0.0	0	0.0	3	1.6	1	3.1	0.200	0	0.0	0	0.0	4	1.1	> 0.900
	Relatives	0	0.0	1	0.8	1	0.5	1	3.1	0.400	0	0.0	1	9.1	2	0.6	0.100
	Friends	0	0.0	0	0.0	1	0.5	0	0.0	> 0.900	0	0.0	0.0	0.0	1	0.3	> 0.900
	Teachers	1	2.3	1	0.8	4	2.1	2	6.3	0.200	1	3.7	0	0.0	7	2.0	0.600
	Medical staff	0	0.0	3	2.3	4	2.1	5	15.6	**0.005**	0	0.0	0	0.0	12	3.4	> 0.900
	Do not remember	0	0.0	2	1.6	0	0.0	0	0.0	0.500	0	0.0	0	0.0	2	0.6	> 0.900
	Other	0	0.0	0	0.0	3	1.6	0	0.0	0.600	0	0.0	0	0.0	3	0.8	> 0.900

Note: Table 5 illustrates that older age, having epilepsy and receiving seizure first aid training are linked to greater knowledge of seizure first aid. Additionally, having epilepsy is associated with a more positive attitude towards seizure first aid.

Bold represents a statistically significant *p*-value ≤ 0.05.

However, not all studies have found a direct correlation between age and knowledge levels. A study among schoolteachers in Jeddah, Saudi Arabia, reported no significant association between knowledge scores and age, suggesting that factors such as qualifications and prior exposure to seizures might play a more influential role.^31^

A statistically significant correlation was observed between having epilepsy and knowledge scores. Most individuals with epilepsy scored in the average to below-average category, while more patients without epilepsy were in the knowledgeable category. This finding aligns with existing research, which suggests that individuals with epilepsy often have limited knowledge about their condition.

A study assessing epilepsy patients’ understanding of their disorder found that neither age, duration of epilepsy nor educational background significantly correlated with their knowledge levels.^32^ Similarly, a study at Jordan University Hospital reported an average correct response rate of only 48% on the Epilepsy Knowledge Profile-General scale, highlighting a significant knowledge gap among patients regardless of factors such as seizure type, duration of epilepsy or source of information.^33^ These findings suggest that having epilepsy does not necessarily equate to a higher level of knowledge about the condition. This unexpected trend in the present study may be attributed to the general lack of awareness among epilepsy patients.

A significant correlation was observed between having received seizure first aid training and knowledge scores. Most participants who had undergone formal training demonstrated higher knowledge scores, reinforcing the effectiveness of structured educational interventions. Interestingly, some individuals without formal training also achieved high knowledge scores, suggesting that additional factors may influence awareness.

Similar patterns have been reported in previous studies. For instance, research among schoolteachers in Northwestern Nigeria found that despite generally low knowledge and persistent misconceptions about epilepsy, higher education levels were significantly associated with improved knowledge and more positive attitudes towards students with epilepsy.^34^ A study among medical students in South India also revealed inadequate first aid knowledge, underscoring the need for formal training to improve these skills.^35^ Moreover, a comparative study of urban and rural schoolteachers in Nigeria showed that, although knowledge and attitudes were generally poor, those with higher education levels exhibited better understanding and perceptions of seizure disorders.^36^

These findings highlight that while formal training is essential, other factors, such as educational attainment and informal exposure, may also contribute meaningfully to acquiring seizure-related knowledge.

In our study, the high scores observed among individuals without formal training may be explained by informal learning gained through personal experiences, social media or witnessing seizure episodes firsthand. Further research exploring these informal knowledge sources in greater detail could offer valuable insights into how people acquire seizure first aid knowledge outside formal training.

### Attitude

Most of our participants agree that people with epilepsy should notify their partners before marriage and that they can have children. These views contrast with the findings of Zhao et al., where it was reported that a majority of the study population would disagree with the notion of their child marrying an individual with epilepsy.^30^ Our participants also believed that individuals with epilepsy can attend regular schools and lead normal lives. This observation aligns with the findings reported by Zhao et al.^30^ The majority of participants stated that people with epilepsy should be able to live normal lives, and most indicated they would not feel embarrassed if a family member had epilepsy. Furthermore, most participants disagreed with the statements suggesting that children with epilepsy should attend special schools or be prohibited from marrying, again consistent with Zhao et al.’s findings.^30^

A significant finding in our study was that individuals with epilepsy demonstrated a positive attitude towards their condition. This can be attributed to their experiences, which lead to a greater understanding and compassion. Interestingly, the majority of participants without epilepsy also exhibited positive attitudes. While this is encouraging, it is possible that social desirability bias influenced responses, with participants selecting answers they perceived as acceptable to align with societal expectations. This raises important considerations regarding the stigma surrounding epilepsy. This finding is supported by a study in Southwest Ethiopia, which found that higher educational status was significantly associated with more positive attitudes.^37^ Similarly, research in the United Kingdom has shown that, despite improvements in public attitudes towards epilepsy over time, stigma persists and continues to impact the social identity of people with epilepsy, especially in professional and social contexts.^38^ In South Korea, public education campaigns led to more positive self-reported attitudes towards epilepsy. Yet, many misconceptions and negative perceptions remained, indicating that implicit biases and stigma can persist even when explicit attitudes appear favourable.^39^

Our study found no correlation between educational level and participants’ knowledge or attitudes towards epilepsy and seizure first aid (also see Table 5). This suggests that formal education alone does not necessarily contribute to a deeper understanding of epilepsy. Rather, factors such as personal experience, exposure to individuals with epilepsy and access to relevant health information may play a more significant role in shaping awareness and perceptions. The lack of a strong relationship between education and epilepsy-related knowledge may be because of the limited integration of epilepsy education into general curricula. Additionally, social and cultural influences likely shape attitudes towards epilepsy more than formal education does. Findings from a study conducted among teachers in Saudi Arabia suggest otherwise. AlMuslim et al. found a significant correlation between educational level and epilepsy knowledge, with teachers with higher academic qualifications demonstrating a better understanding of epilepsy and seizure first aid.^40^ This may be explained by the specific nature of the study population; teachers are more likely to encounter students with epilepsy, which could lead to greater exposure to epilepsy-related training and resources. Additionally, teachers may have better access to structured health education programmes than the general population, which could enhance their knowledge levels. In contrast, our study sampled a more diverse population, where access to epilepsy-related education may be inconsistent and dependent on individual experiences rather than formal schooling. These findings highlight the need for targeted epilepsy awareness programmes that extend beyond professional settings, ensuring that knowledge is accessible to the broader community regardless of educational background.

## Limitations

A key limitation of the study is the use of convenience sampling, which, along with the relatively small and homogeneous sample, may not accurately reflect the broader Bloemfontein population, limiting the generalisability of the findings. A more diverse sample, including participants from various educational, socioeconomic and geographic backgrounds, particularly from rural or underserved areas, could offer a more comprehensive understanding of public knowledge and attitudes towards epilepsy. Additionally, the study’s geographic focus on Bloemfontein may not accurately reflect perspectives from other regions, where cultural, religious and social norms surrounding epilepsy differ. As these factors can significantly influence awareness and attitudes, future research should consider regional variations in beliefs and access to epilepsy education and health care.

Another limitation of the study is the potential for response bias. Social desirability bias may have influenced participants to provide answers they perceived as socially acceptable, especially concerning attitudes towards epilepsy, thereby potentially overstating positive views. In addition, the reliance on self-reported data introduces the risk of recall bias, as participants may have over- or under-estimated their knowledge or past experiences. To enhance the accuracy of future research, incorporating objective assessments such as scenario-based questions or practical demonstrations of seizure first aid would provide a more reliable evaluation of actual knowledge and skills.

## Recommendations

Our study highlights a significant gap in public knowledge about epilepsy and seizure first aid, contributing to stigma and inappropriate responses. To address this, concise and culturally appropriate pamphlets and posters should be distributed nationwide in clinics and health facilities.

Given increasing digital literacy, online platforms and mobile-accessible resources should also be developed to share reliable information, particularly targeting youth and urban populations. Combining traditional and digital outreach can improve public awareness, reduce stigma and equip individuals with essential seizure first aid skills.

Alternatively, programmes should be put in place targeting schools to educate the younger generation, as they were found to be the least knowledgeable about epilepsy, ensuring that religious and spiritual leaders are involved to effectively address the issue of misconceptions.

## Conclusions

The results of our study revealed that the attendees of the Gateway Clinic were knowledgeable and had positive attitudes towards epilepsy and seizure first aid. Statistically significant associations were found between participants’ knowledge and attitudes towards epilepsy and the following factors: age, having epilepsy, knowing someone with epilepsy and learning about seizure first aid.

While the majority of the study population fell into the ‘knowledgeable’ category, gaps in knowledge persist, highlighting the need for public education. These findings suggest a promising foundation for broader societal acceptance of epilepsy, which can be further enhanced through culturally sensitive, targeted health education campaigns.

Community engagement and accessible platforms are crucial for addressing misconceptions, reducing stigma and equipping the public with lifesaving seizure first aid knowledge.
